# Seroprevalence of Zika virus in pregnant women from central Thailand

**DOI:** 10.1371/journal.pone.0257205

**Published:** 2021-09-13

**Authors:** Chayawat Phatihattakorn, Artit Wongsa, Kirakorn Pongpan, Sanitra Anuwuthinawin, Sakita Mungmanthong, Manthana Wongprasert, Boonrat Tassaneetrithep

**Affiliations:** 1 Department of Obstetrics and Gynecology, Faculty of Medicine Siriraj Hospital, Mahidol University, Bangkok, Thailand; 2 Center of Research Excellence in Immunoregulation, Faculty of Medicine Siriraj Hospital, Mahidol University, Bangkok, Thailand; 3 Division of Obstetrics and Gynecology Nursing, Department of Nursing, Siriraj Hospital, Bangkok, Thailand; CEA, FRANCE

## Abstract

Zika virus (ZKV) infection in a pregnant woman, especially during the first trimester, often results in congenital anomalies. However, the pathogenic mechanism is unknown and one-third of ZKV infected pregnancies are asymptomatic. Neutralizing antibodies against ZKV has been reported in 70% of Thai adults, but the prevalence among pregnant women is unknown. Currently, vaccines and specific treatments for ZKV are under development. A better understanding of the immune status of pregnant women will increase the success of effective prevention guidelines. The prevalence of ZKV infection in pregnant women in antenatal care clinics was investigated during the rainy season from May to October 2019 at Siriraj Hospital, Bangkok, Thailand. We recruited 650 pregnant women (39.42% first, 52.26% second and 7.36% third trimester) and found that 30.77% had ZKV-specific IgG, and 39.81% had neutralizing antibodies (nAb) against ZKV (titer ≥10). Specific and neutralizing antibody levels varied by maternal age, trimester, and month. We further characterized the cross-reaction between ZKV and the four Dengue virus (DENV) serotypes by focused reduction neutralization test (FRNT) and found that cross-reactions were common. In conclusion, about 60% of pregnant women who living in central Thailand may be at risk of ZKV infection due to the absence of neutralizing antibodies against ZKV. The functions of cross-reactive antibodies between related viral genotypes require further study. These findings have implications for health care monitoring in pregnant women including determining the risk of ZKV infection, assisting the development of a flavivirus vaccine, and informing the development of preventative health policies.

## Introduction

Zika virus (ZKV) is an RNA virus belonging to the genus *Flavivirus* and family *Flaviviridae* that includes several mosquito-borne viruses such as Dengue (DENV), Japanese encephalitis (JEV), West Nile (WNV), and Yellow fever (YFV) viruses [1]. *Aedes spp* mosquitoes are the primary vector and major transmission route of ZKV to humans [2]. Transmission via sexual intercourse, blood transfusion, laboratory accidents and vertical transmission has been documented [2, 3]. ZKV typically causes a mild, self-limited illness with fever, headache, generalized maculopapular rash, conjunctivitis, and arthralgia [4]. In adults, ZKV is associated with severe complications such as Guillain-Barré syndrome (GBS) and meningoencephalitis [5, 6]. Clinical diagnosis is difficult because Zika infection often presents with symptoms similar to those of other mosquito-borne viruses, including DENV and Chikungunya virus (CHIKV) infections.

Many studies have reported fetal abnormalities in ZKV infected pregnant women, especially during their first trimester [7–9]. The abnormalities are generally found in the central nervous system (CNS) such as ventriculomegaly, ventricular/vermis agenesis, abnormal middle cerebral artery flow, visual impairment and microcephaly [10–12]. Evidence of morbidity and mortality of fetus in ZKV infected pregnant women have been reported [13].

ZKV is endemic in tropical areas, as are other mosquito-borne arboviruses in the genus *Flavivirus* and *Togavirus* [4, 14, 15]. However, Zika infected cases have been observed sporadically worldwide due to the multiple transmission routes [16]. Thailand has reported ZKV infections since 2012 [17, 18]. In 2017, Sornjai and colleagues reported that nearly 70% of Thai adults had neutralizing antibodies against ZKV [19]. A study of febrile patients in Thailand found that antibodies against mosquito-borne *Flaviviridae* may cross-react with *Togaviridae* viruses [20]. In an animal model, the presence of neutralizing antibodies can prevent fetal anomaly [21]. Pre-existing neutralizing flavivirus antibodies may also increase disease severity through antibody-dependent enhancement [22]. Therefore, we studied the prevalence of antibodies to ZKV in pregnant Thai women to better understand the risk of infection.

## Materials & methods

### Enrollment of pregnant women

This study was approved by the Siriraj Institutional Review Board (si061/2019). Pregnant women in the antenatal clinic (ANC) at Siriraj Hospital were invited to join the study according the IRB-approved recruitment protocol. The sample size was calculated based on prevalence of asymptomatic ZKV infected Thai pregnant women 0.8% [23], 95% confidence, and margin of error  =  0.07. After providing consent, three milliliters of blood were collected, centrifuged, and the serum was frozen at -80 ^o^C until testing.

### ZKV-specific IgG detection

The level of the ZKV-specific IgG was measured using a commercial Anti-Zika Virus IgG ELISA EI 2668–9601 G (Euroimmun, Lübeck, Germany) according to the manufacturer’s instructions [24]. Optical density (OD) is measured in duplicate, and data is interpreted as follows; OD<0.9 is negative, OD 0.9–1.1 is equivocal, and OD>1.1 is positive. Participants who have tested positive for ZKV-specific IgG and returned to the hospital for routine follow-up of ANC were required to provide a second blood sample (2–4 weeks after the initial collection). Without the results of RNA or ZKV-specific IgM detection, a four-fold rise in ZKV-specific IgG levels between paired samples, one of the indications of recent infection [25] was utilized to determine the infection status.

### Virus preparation

Zika virus and the four dengue virus serotypes were propagated in C6/36 cells (kindly provided from Dr. Thaneeya Duangchinda, National Center for Genetic Engineering and Biotechnology, Thailand) with Leibovitz’s L-15 medium (Gibco, Carlsbad, USA), 1.5% fetal bovine serum (FBS, Gibco, Carlsbad, USA), 10% Tryptose Phosphate Broth (TPB, Merck Millipore, Billerica, USA), 2 mM of L-Glutamine, 100 units/mL of Penicillin, and 100 μg/mL of Streptomycin (Gibco, Carlsbad, USA) at 28°C. After 5, 7, and 9 days of post-infection, supernatant was collected and stored at -80 ^o^C until testing.

Viral concentration was determined using focus forming assay. Briefly, Vero cells (kindly provided from Prof. Pilaipan Puthavathana, Faculty of Medical Technology, Mahidol University, Thailand) with Earle’s minimal essential medium (EMEM, Gibco, Carlsbad, USA), 10%FBS, 100 units/mL of Penicillin, and 100 μg/mL of Streptomycin were seeded in a 96-well plate and left overnight in 5% CO_2_ inside a 37°C incubator. The propagated virus with a dilution range of 10^−2^ to 10^−7^ was transferred to Vero cells. 3% Carboxymethylcellulose (CMC, Merck Millipore, Billerica, USA) was added after 1 hour incubation and continued incubating at 37°C for two or three days (depending on viral serotypes). Finally, the cells were fixed and stained with a pan-flavivirus monoclonal antibody (clone 4G2, kindly provided from Dr. Chunya Puttikhunt, National Center for Genetic Engineering and Biotechnology, Thailand) as the primary antibody; horseradish peroxidase (HRP) goat anti-mouse IgG (Agilent Technologies, CA, USA) as a secondary antibody, and 3,3′-Diaminobenzidine (DAB, Merck Millipore, Billerica, USA) as substrate. The plaque number was calculated in focus forming units (FFU)/ml.

### Neutralizing antibody titration

Sera were inactivated at 56°C for 30 minutes and diluted 2-fold for six titers and mixed with 80–100 FFU of a virus. The serum-virus complex was transferred into monolayered Vero cells in a 96-well plate and incubated for 1 hour. 3% CMC was added and incubated for two or three days. Finally, cells were fixed and stained with 4G2 antibody, HRP goat anti-mouse IgG, and DAB. A final serum dilution that reduced > 50% of the viral foci was presented as the neutralizing antibody (nAb) titer.

### Data analysis

The difference in specific anti-ZKV IgG and nAb between groups was compared by ANOVA, a *p-value* <0.05 was regarded as significant. The correlation of specific IgG and nAb titer was determined by Spearman rank correlation. Correlation plot using ggplot2 package. Heatmap using ggplot2 package. A t-distributed stochastic neighbor embedding (t-SNE) plot using the Rtsne package. Data analysis was conducted using GraphPad Prism 9 (Version 9.0.1 (151), San Diego, CA, USA) and RStudio (Version 1.2.5033, Boston, MA, USA).

## Results

### Demographic data of pregnant women

Six hundred and fifty pregnant women were enrolled in the study between May and October 2019. Four hundred and seventy-three of the participants live in central Thailand (S1 Table). The mean age of participants is 29.46 years old (range 18–45). The first, second, and third-trimester proportions were 39.54%, 53.08%, and 7.38%, respectively (Table 1). Only two percent of participants were able to recall the signs and symptoms of a ZKV infection in the preceding one or two months (Table 1).

**Table 1:** Demographic data of pregnant women.

Characteristics	Mean	Range	Frequency, (%)
Age (year old)	29.46	18–45	
Gestational (weeks)	18.15	0–40	
First trimester (wks.)	9.54	0–14	257 (39.54)
Second trimester (wks.)	22.57	15–28	345 (53.08)
Third trimester (wks.)	32.64	29–40	48 (7.38)
Sign of symptom			
Fever	-	-	14 (2.15)
Rash	-	-	3 (0.46)
Conjunctivitis	-	-	0 (0.00)
Couple			
Age (year old)	31.55	14–63	
Sign of symptom			
Fever	-	-	11(1.69)
Rash	-	-	0 (0.00)
Conjunctivitis	-	-	2 (0.31)

### Serology status of pregnant women

**Fig 1:**
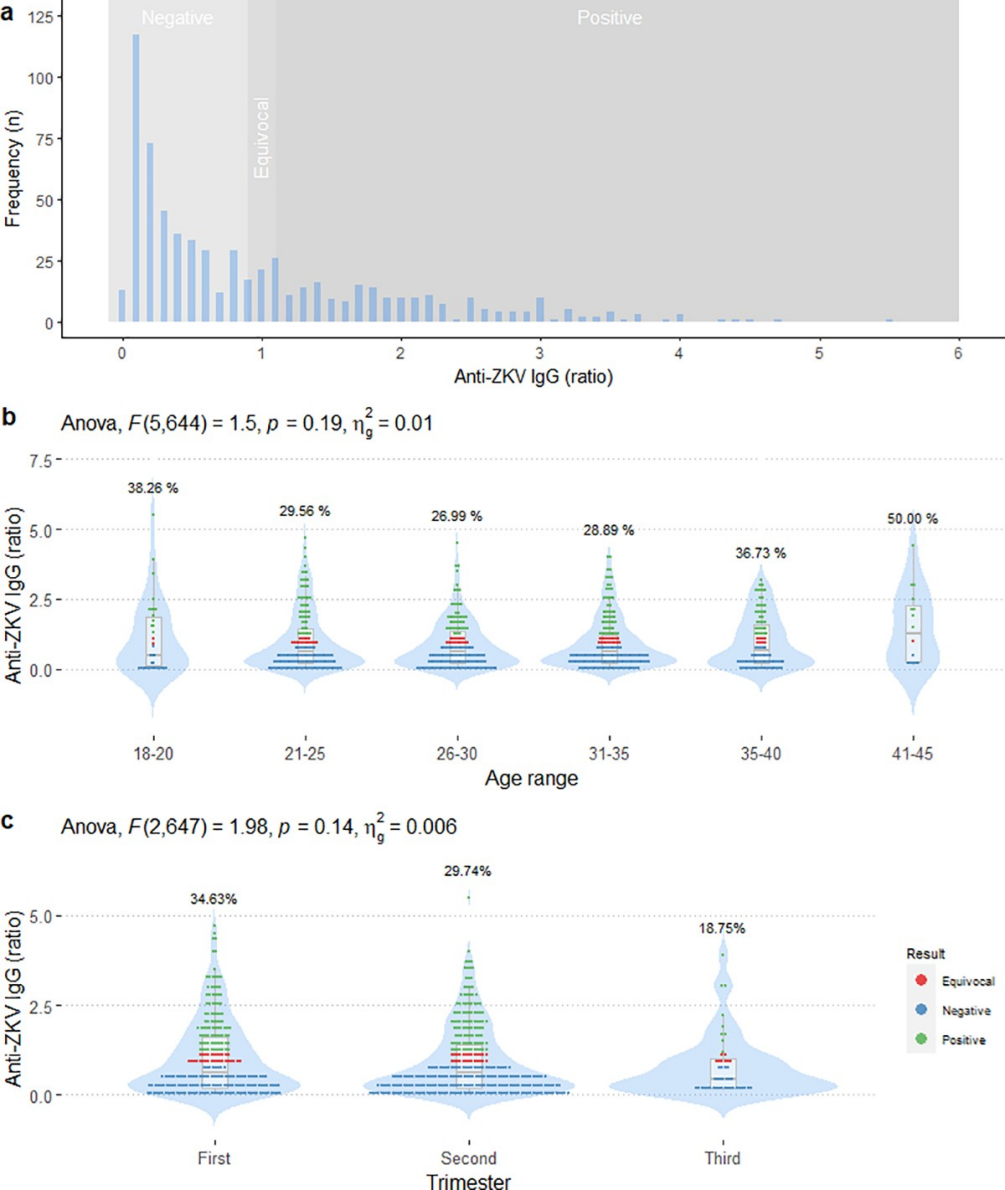
The Zika virus-specific IgG in pregnant women. The distribution of Zika virus-specific IgG in pregnant women (a). ZKV-specific IgG by age range (b) or trimester (c).

Two hundred (30.77%) women were positive for ZKV-specific IgG, 62 (9.54%) were equivocal, and 388 (59.69%) were negative (Fig 1A). ZKV-specific IgG were present in 26% to 50% of participants in each of six maternal age groups (Fig 1B). Eighty-nine first trimester participants (34.63%) were positive for ZKV-specific IgG. The second-trimester group had 102 (29.74%) positive, and the third-trimester group had 9 (18.75%) positive (Fig 1C). There was no statistically significant difference in the proportion of participants with ZKV-specific IgG by age group or by trimester.

One hundred and fifty-six ZKV-specific IgG positive participants agreed to provide a second blood sample 2–4 weeks after the initial collection. No participant had a four-fold rise of ZKV-specific IgG, but 43/156 participants (27.56%) had a less than four-fold increase in titer. Seventy-seven participants (49.36%) showed a reduction in ZKV-specific IgG levels from the initial visit. While, the level of ZKV-specific IgG remained unchanged in 36 participants (23.08%) (S1 Fig).

### Neutralizing antibody level of Zika and Dengue virus

**Fig 2:**
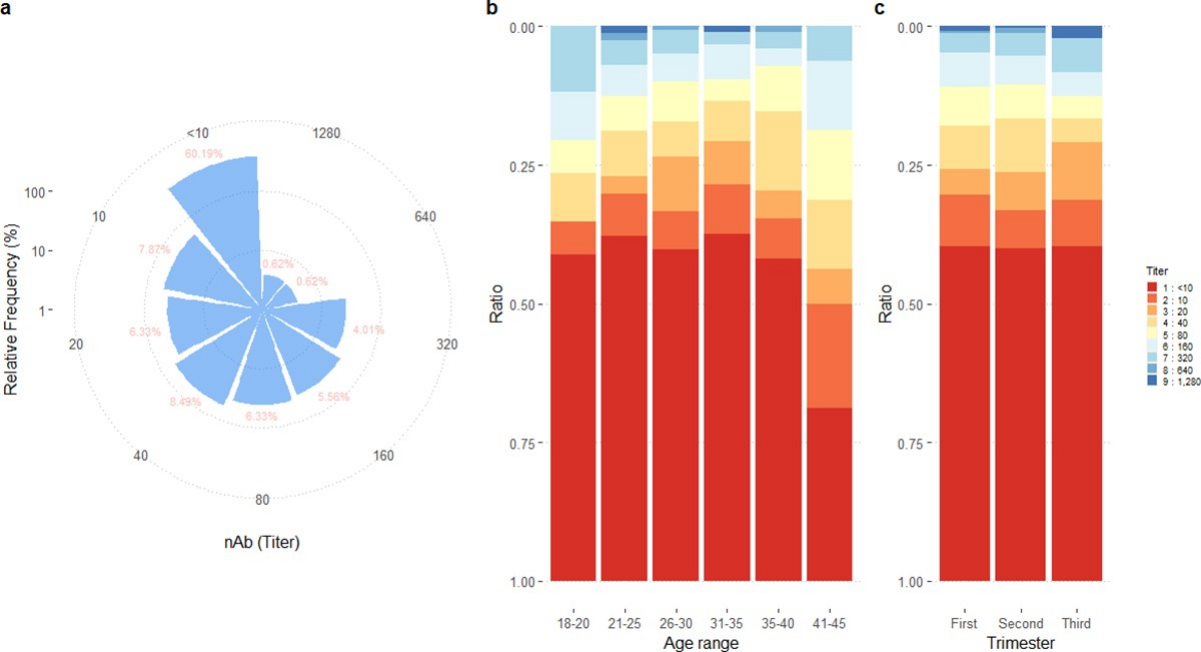
The titer of nAb in pregnancies. Serum was screened for the ZKV-nAb using FRNT (a). The results of nAb titer from all pregnant women were divided by age group (b) and trimester (c).

Focused reduction neutralization assay is the gold standard for nAb level assessment. Of 648 (99.69%) samples with sufficient material to be processed, 390 samples (60.19%) had a nAb titer < 10, while 258 samples (39.81%) had a titer ≥ 10. Sixty percent of participants had low ZKV-nAb titer (Fig 2A). The proportions of women with a nAb titer <10 were similar between age groups (18–20: 58.82%, 21–25: 62.26%, 26–30: 59.87%, 31–35: 62.57%, 35–40: 58.16%). Interestingly, the lowest proportion of nAb titer <10 (31.25%) was in the 41 to 45-year age group. There was no statistically significant difference in the proportion of participants with nAb by maternal age (Fig 2B) or trimester of pregnancy (Fig 2C).

**Fig 3:**
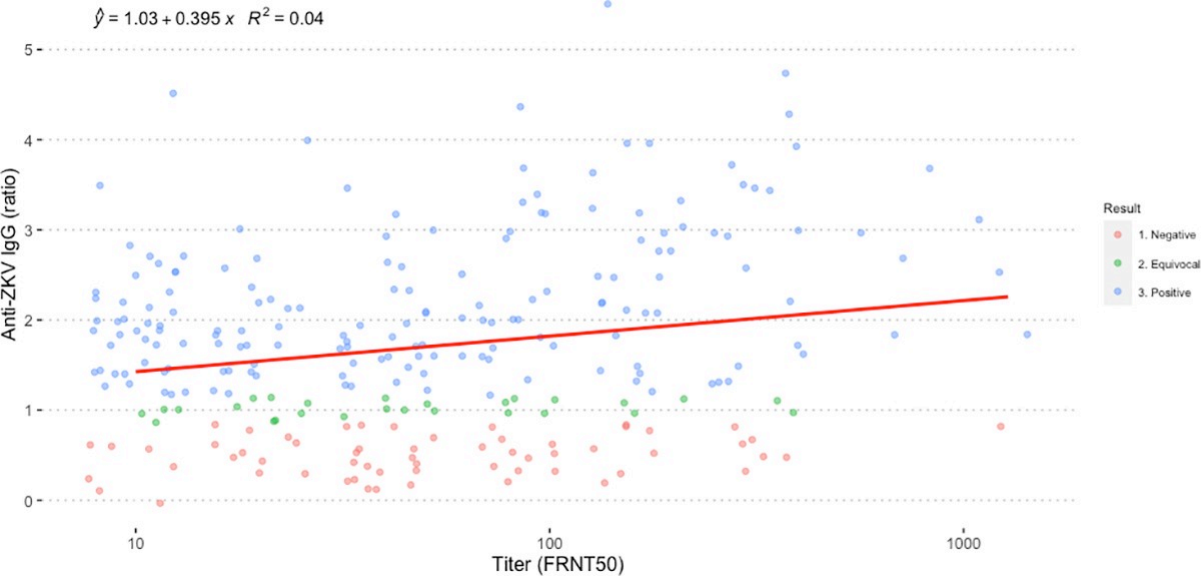
The correlation of ZKV-nAb titer and ZKV-specific IgG. The correlation graph and equation were generated using ggplot2 package in R. nAb titer (x-axis) and ZKV-specific IgG (y-axis).

The 258 samples with nAb titer ≥ 10 were used to evaluate a potential correlation between ZKV nAb and the level of ZKV-specific IgG (Fig 3). There was no correlation between the level of specific IgG and nAb titer.

**Fig 4:**
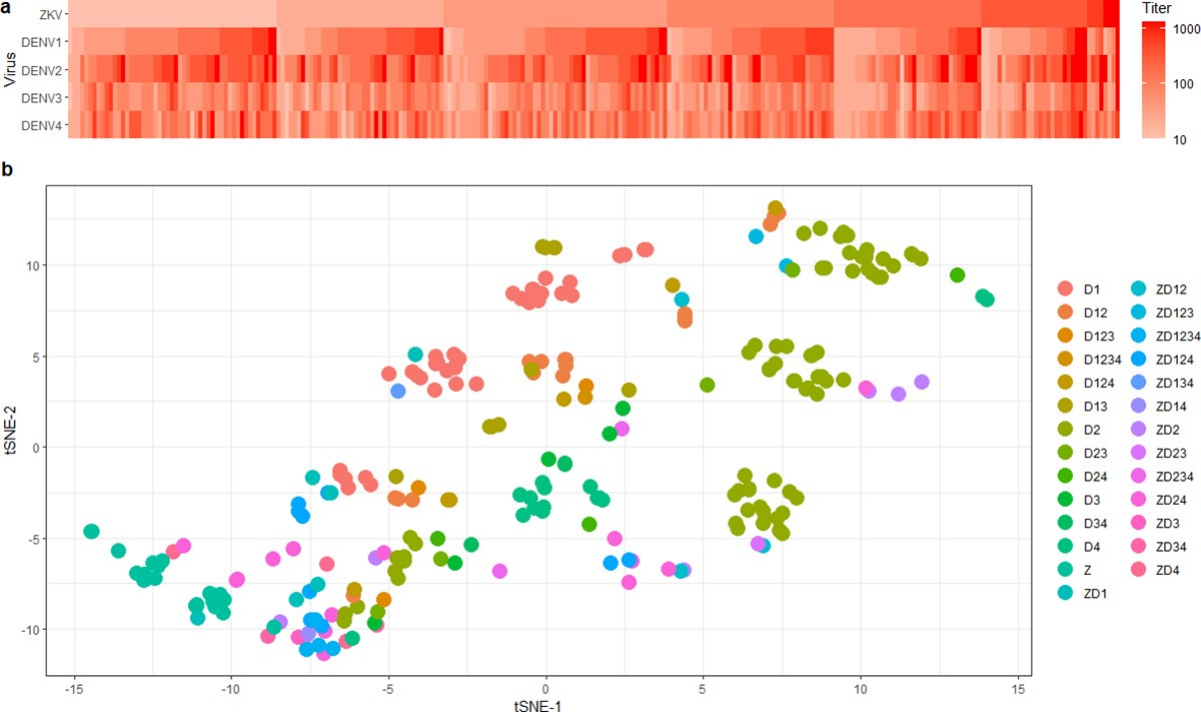
The relationship of nAb titer for ZKV and DENV. All nAb titer for ZKV and each DENV serotype were shown in a heatmap (a) using ggplot2 package. The nAb titer against ZKV or each DENV serotype were visualized in a t-distributed stochastic neighbor embedding (t-SNE) plot (b) using Rtsne package.

Samples that contained nAb titer ≥ 10 were measured against all four serotypes of DENV. An irregular pattern in the heatmap was observed (Fig 4A), suggesting that cross-reactions between ZKV and each serotype of DENV were common. The ZKV-nAb was detected in 8.14% (21/258) of samples, while 23.25% (58/258) had pan nAb against ZKV and only one or more DENV serotype. In comparison, 69.38% (179/258) showed pan-specific nAb against all four DENV serotypes. DENV-serotype-specific nAb were identified in 50.77% (131/258) including 14.34% (37/258) for serotype 1; 27.91% (72/258) for serotype 2; 1.94% (5/258) for serotype 3, and 6.59% (17/258) for serotype 4 (S2 Fig). A statistical positive correlation of nAb titer between ZKV vs DENV 2 and ZKV vs DENV 3 were observed. Dimensionality reduction was used to visualize the nAb titers which were clustered in serotype-specific groups (Fig 4B).

### The dynamic of specific IgG and nAb in the raining season

**Fig 5:**
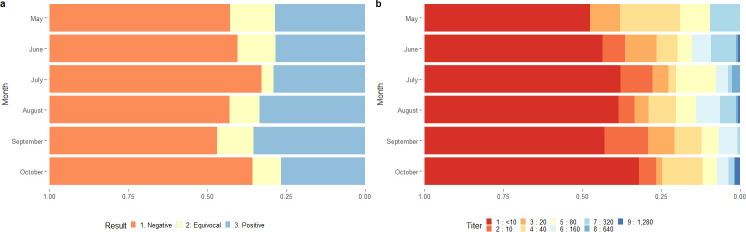
Frequency of ZKV-specific IgG and nAb titer in pregnant women by month. Sera of all participants were screened for ZKV-specific IgG and nAb titer. ZKV-specific IgG (a) and nAb titer (b) were separate by month.

All samples were collected during the rainy season (May-Oct) because mosquito-borne diseases are prominent during this period. The following are the result of ZKV-specific IgG of pregnant women who visit ANC: in May, 6/21(28.57%) were ZKV IgG positive; in June 43/151 (28.48%) were positive; in July 23/79 (29.11%) were positive; in August 53/158 (33.54%) were positive; in September 46/130 (35.38%) were positive, and in October 29/109 (26.60%) were positive (Fig 5A). We did not observe a statistically significant difference in the proportion of participants with nAb titer ≥ 10 between months (Fig 5B).

## Discussion

Pregnant woman may be more susceptible to viral infection, their illnesses may be more severe, and prenatal infections may result in fetal anomalies [26]. Vaccination against serious infectious diseases such as Influenza, Tetanus, Diphtheria, and Pertussis in pregnant women is recommended worldwide [27]. However, currently there is no approved vaccine to prevent ZKV infection and no specific treatments [28]. Previous infection with ZKV can induce long-term immunological memory [29]. ZKV-specific or neutralizing antibodies are part of adaptive immunity that recognizes ZKV antigens and protects against infection [30].

We detected ZKV-specific IgG in 30.77% of 650 pregnant women. This suggests that nearly 70% of our participants had not been exposed to the ZKV. The positivity rate of ZKV-specific IgG was 2.89 times lower than that of pregnant women in Colombia during a ZKV epidemic [31]. There were no statistically significant differences in antibody prevalence between maternal age groups. Different rates of ZKV-specific IgG between gestational age groups were observed. However, we were unable to determine the recent infection because we did not observe any individuals with signs of infection or a four-fold rise in ZKV-specific IgG levels.

Sixty percent of our participants had an absent or low level (titer < 10) of ZKV-nAb. However, the percentage of our participants with nAb ≥ 10 was 1.75 times less than in a healthy adult population in Thailand in 2017 [19]. The disparity in results might be attributed to population differences, year of enrollment, participant living region, and antibody waning [32, 33]. Neutralizing antibody titers varied across maternal and gestational age. The age of persons living in the epidemic area can influence their risks of exposure to the virus [34], yet the group of pregnant women (n = 16) aged 41 to 45 in our study had the lowest ratio of nAb titer < 10.

The clinical outcome of a fetus of ZKV infected women is also related to gestational age at the time of infection and the stage of brain development [35]. Absent or insufficient nAb against ZKV may increase susceptibility to infection [36], while pre-existing nAb is able to prevent viral infection or reduce disease severity [37]. Thus, most of the participants in our study were at risk of ZKV infection. We did not detect an association between ZKV-specific IgG and nAb levels. Therefore, both ZKV-specific IgG and nAb should be considered to avoid underestimating the risk of infection. The combination of two assays increases sensitivity and specificity [38]. The presence of cross-reactivity affects the result of ZKV-specific IgG [29]. DENV-specific antibodies might interfere with the result of nAb titer against ZKV. One study reported that pre-existing nAb against a broad spectrum of Flavivirus are protective against poor clinical outcomes [39]. In addition, the individual who have antibodies against the envelope protein domain I/II or fusion loop epitope may be more easily infected with ZKV due to the effects of antibody dependent enhancement (ADE) [40–44].

We found pan-nAb against ZKV and DENV in 23.25% of women with ZKV-nAb at a titer ≥ 10. These cross-protective antibodies could be the result of multiple infections or epitope homology [39, 45]. This finding suggests that defining of common epitopes are a potential approach for universal vaccine or pan protective-nAb development [46]. we found that the ratio of DENV serotype-specific nAb was the same as the ratio of Dengue serotypes in infected individuals in Thailand from 2016 to 2019 [47].

The seroprevalence rate at the end of the rainy season was higher than in the early season. An increasing abundance of mosquitos in the raining season might increase the risk of exposure [48, 49]. Ninety-eight percent of participants were unable to recall any ZKV-related symptoms before enrollment. ZKV infection typically results in mild or asymptomatic illness, which is difficult to diagnose without laboratory confirmation [50]. Severe complications in the fetus have been observed in asymptomatic maternal ZKV infections [51]. Therefore, education on ZKV infection and protective behaviors should be included in prenatal education, especially in the epidemic area.

## Conclusion

Education on ZKV infection and protective behaviors should be included in prenatal education, especially in endemic areas. Understanding immune-protective status, especially humoral immune responses, may help to improve health policies for pregnant women. Routine screening of ZKV-nAb titers or the implementation of routine rapid antibody testing during antenatal care should be considered. Pregnant women should be advised to carefully monitor themselves for signs of infection and to promptly seek medical attention. Expanded surveillance for ZKV infection is a public health priority. Finally, further research on the impact of pre-existing humoral immune responses and the role of antibody dependent enhancement is warranted.

## Supporting information

S1 Table City of pregnancies residence. (DOCX)

S1 Fig Variation of specific zika virus IgG antibody in pregnant women. Sera of all volunteers from the initial visit (blue) and second visit (red) were screened for Zika virus IgG using ELISA.
(TIF)

S2 Fig The serotype-specific nAb in pregnant women. The serotype-specific nAb were demonstrated in an upset plot using UpsetR package.
(TIF)

S1 Data (XLSX)
