# Correction: Seroprevalence of Zika virus in pregnant women from central Thailand

**DOI:** 10.1371/journal.pone.0261598

**Published:** 2021-12-14

**Authors:** Chayawat Phatihattakorn, Artit Wongsa, Kirakorn Pongpan, Sanitra Anuwutnavin, Sakita Moungmaithong, Manthana Wongprasert, Boonrat Tassaneetrithep

The fourth author’s name is spelled incorrectly. The correct name is: Sanitra Anuwutnavin. The fifth author’s name is spelled incorrectly. The correct name is: Sakita Moungmaithong. The correct citation is: Phatihattakorn C, Wongsa A, Pongpan K, Anuwutnavin S, Moungmaithong S, Wongprasert M, et al. (2021) Seroprevalence of Zika virus in pregnant women from central Thailand. PLoS ONE 16(9): e0257205. https://doi.org/10.1371/journal.pone.0257205
