# Supplementary material for: Seroprevalence of Zika virus in pregnant women from central Thailand
Source: PLoS One. 2021 Sep 13;16(9):e0257205. doi: 10.1371/journal.pone.0257205 (PMC8437263; doi:10.1371/journal.pone.0257205)
Supplement: S1 Table — (DOCX) [file pone.0257205.s001.docx]

S1 Table. City of pregnancies residence

| Province | Positive  n (%) | Equivocal  n (%) | Negative  n (%) | Total |
| --- | --- | --- | --- | --- |
| Bangkok | 149 (31.5) | 45 (9.51) | 279 (58.99) | 473 |
| Chon Buri | 0 (0) | 0 (0) | 2 (100) | 2 |
| Nakhon Pathom | 9 (22.50) | 4 (10.00) | 27 (67.50) | 40 |
| Nonthaburi | 31 (32.29) | 10 (10.42) | 55 (57.29) | 96 |
| Pathum Thani | 1 (25.00) | 0 (0) | 3 (75.00) | 4 |
| Phra Nakhon Si Ayutthaya | 0 (0) | 0 (0) | 1 (100) | 1 |
| Ratchaburi | 0 (0) | 0 (0) | 1 (100) | 1 |
| Samut Prakan | 5 (45.45) | 1 (9.10) | 5 (45.45) | 11 |
| Samut Sakhon | 5 (26.32) | 2 (10.53) | 12 (63.16) | 19 |
| Samut Songkhram | 0 (0) | 0 (0) | 1 (100) | 1 |
| Suphan Buri | 0 (0) | 0 (0) | 1 (100) | 1 |
|  | 200 | 62 | 387 | 649 |
