# Supplementary figures and images for: Seroprevalence of Zika virus in pregnant women from central Thailand
Source: PLoS One. 2021 Sep 13;16(9):e0257205. doi: 10.1371/journal.pone.0257205 (PMC8437263; doi:10.1371/journal.pone.0257205)

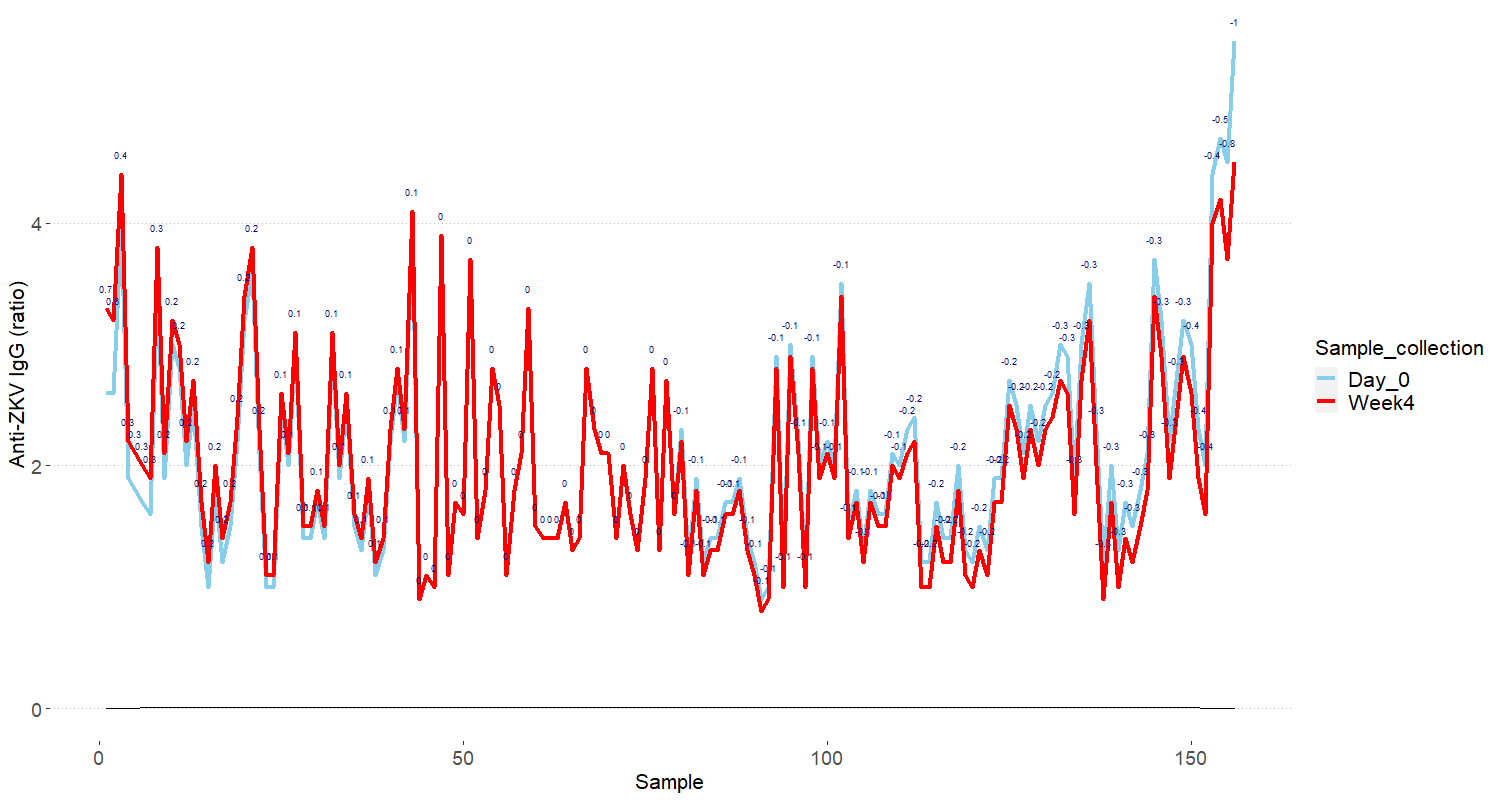

Supplement: S1 Fig — Sera of all volunteers from the initial visit (blue) and second visit (red) were screened for Zika virus IgG using ELISA. (TIF) [file pone.0257205.s002.tif]

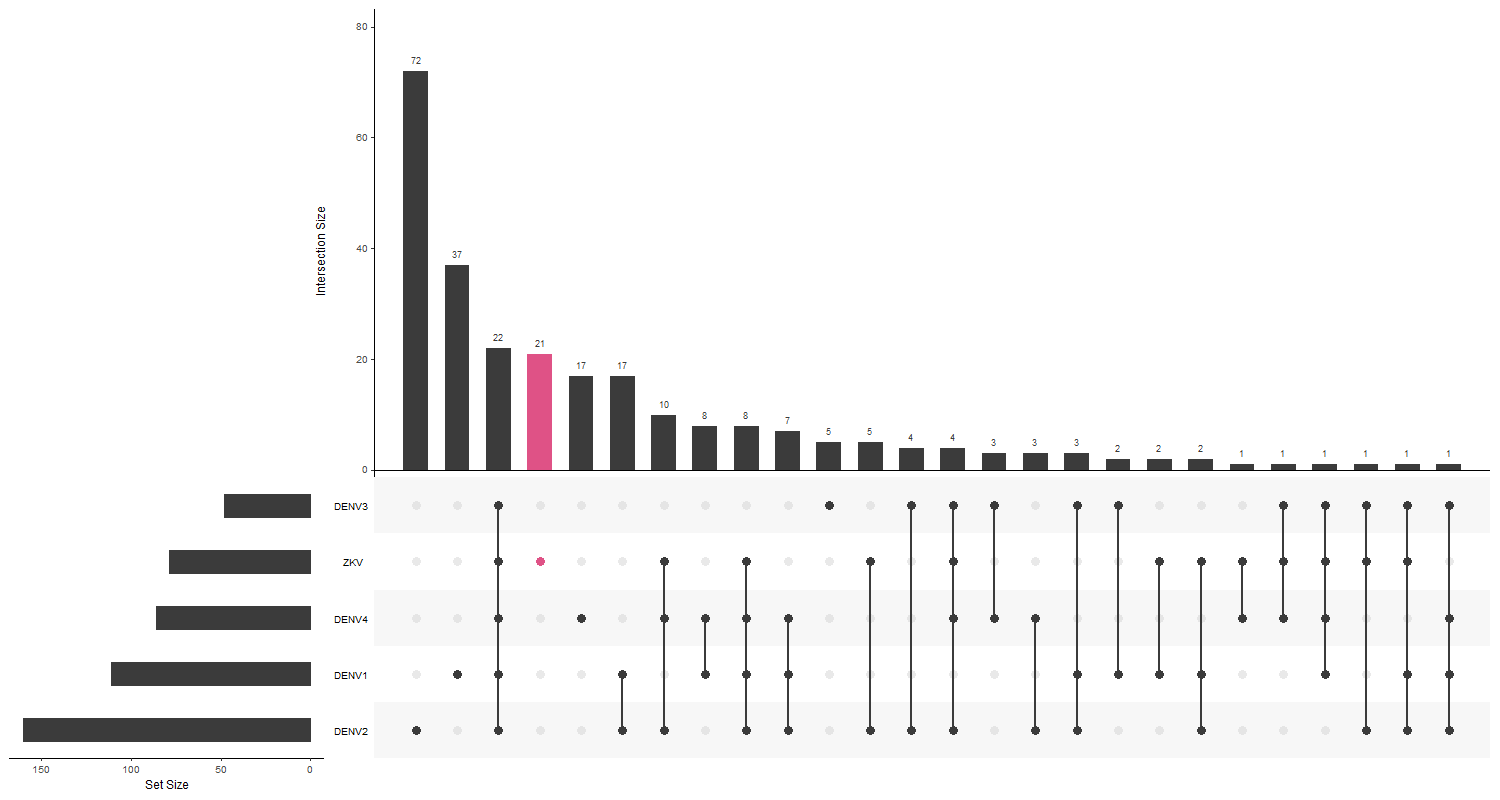

Supplement: S2 Fig — The serotype-specific nAb were demonstrated in an upset plot using UpsetR package. (TIF) [file pone.0257205.s003.tif]
